# Self protein-protein interactions are involved in TPPP/p25 mediated microtubule bundling

**DOI:** 10.1038/srep13242

**Published:** 2015-08-20

**Authors:** Salvatore DeBonis, Emmanuelle Neumann, Dimitrios A. Skoufias

**Affiliations:** 1 Université de Grenoble Alpes, F-38044 Grenoble, France; 2 CNRS, F-38044 Grenoble, France; 3 CEA, IBS, F-38044 Grenoble, France

## Abstract

TPPP/p25 is a microtubule-associated protein, detected in protein inclusions associated with various neurodegenerative diseases. Deletion analysis data show that TPPP/p25 has two microtubule binding sites, both located in intrinsically disordered domains, one at the N-terminal and the other in the C-terminal domain. In copolymerization assays the full-length protein exhibits microtubule stimulation and bundling activity. In contrast, at the same ratio relative to tubulin, truncated forms of TPPP/p25 exhibit either lower or no microtubule stimulation and no bundling activity, suggesting a cooperative phenomenon which is enhanced by the presence of the two binding sites. The binding characteristics of the N- and C-terminally truncated proteins to taxol-stabilized microtubules are similar to the full-length protein. However, the C-terminally truncated TPPP/p25 shows a lower Bmax for microtubule binding, suggesting that it may bind to a site of tubulin that is masked in microtubules. Bimolecular fluorescent complementation assays in cells expressing combinations of various TPPP/p25 fragments, but not that of the central folded domain, resulted in the generation of a fluorescence signal colocalized with perinuclear microtubule bundles insensitive to microtubule inhibitors. The data suggest that the central folded domain of TPPP/p25 following binding to microtubules can drive s homotypic protein-protein interactions leading to bundled microtubules.

Microtubules (MTs) are hollow tubular cytoskeletal filaments of α and β tubulin that play an important role in intracellular processes such as cell morphogenesis, polarity, directional motility, axonal transport and cell division. The functions of MTs are mediated not only by the intrinsic assembly and dynamic properties of tubulin and MTs respectively, but also by their interacting partner proteins. The number of known MT-associated proteins (MAPs) is continuously increasing and encompasses proteins with MT nucleating, assembly, disassembly, stabilizing and severing activity, including MT-end tip binding activity, as well as motor proteins such as kinesins and dyneins which mediate the transport of cargoes along MTs^1^,^2^,^3^,^4^. A small number of MAPs induce MTs to form bundles, including members of the PRC1/MAP65 protein family^5^,^6^,^7^, neuronal MAPs such as tau and MAP2^8^,^9^ and motor proteins such as Eg5 kinesin^10^. MT bundles are encountered in the mitotic central spindle as well as in the midbody during cytokinesis^10^ and in neuronal axons^11^.

TPPP/p25 (Tubulin Polymerization Promoting Protein) is a brain specific protein which binds to tubulin and induces MT bundle formation both *in vitro* and in cells^12^,^13^. TPPP/p25 was first partially co-purified with a tau kinase^14^ and then isolated from bovine brain^12^. TPPP/p25 is expressed specifically in oligodendrocytes, which are essential for the proper development and function of axonal networks in the central nervous system^15^,^16^,^17^. Interestingly, TPPP/p25 was found with α-synuclein in pathological neuronal inclusions such as Lewy bodies, which are major hallmarks for Parkinson’s disease and other synucleinopathies^18^,^19^. TPPP-like proteins identified in diverse eukaryotes have been grouped into a superfamily of TPPP-like proteins that all share amino acid similarity within the central p25 domain^20^. Initial biophysical studies have shown that, like other MAPs, TPPP/p25 has a low helical content and is highly flexible or even disordered^12^,^21^,^22^. Indeed, structural studies on three TPPP-like proteins from different species have revealed a conserved central domain composed of alpha helices flanked by disordered N- and C-terminal domains of variable length^23^,^24^,^25^.

TPPP/p25 was shown to polymerize tubulin into double-walled tubules, polymorphic aggregates or bundle-stabilized MTs^13^. TPPP/p25 co-localizes selectively with the microtubule network in eukaryotic cells causing stabilization of the system; the overexpression of this protein in transfected HeLa cells induces a characteristic protein aggregation reminiscent of the process of aggresome formation^26^. This process may be related to the enrichment of TPPP/p25 in inclusion bodies in the brains of patients afflicted with Parkinson’s disease or other synucleinopathies^18^,^19^,^27^. In addition, the binding of TPPP/p25 to tubulin has been shown to bind *in vitro* and therefore maybe regulated in cells by GTP^21^. In cells, TPPP/p25 targets the microtubule network by blocking mitotic spindle formation without dramatically interfering with any other MT-dependent functions^13^. In addition, at low expression levels, TPPP/p25 dynamically co-localizes with MTs and induces MT bundling and stabilization followed by a subsequent increase in acetylated MTs^28^. At high expression levels, TPPP/p25 induces aberrant MT ultrastructures characterized by “double-walled” MTs and disordered bundles, promoting cell death^26^. Therefore, the physiological function of TPPP/p25 may be to stabilize physiological microtubule ultrastructures (through its MT bundling activity), whereas its upregulation would disorganize the MT cytoskeleton and initiate abnormal protein aggregates such as pathological inclusions^26^.

To date, only a few studies have directly addressed the interactions between TPPP/p25 and MTs at the molecular level. Initially, it was thought that the MT binding properties of TPPP may reside within the central p25 core and/or C-terminal domain, since the shorter, N-terminally truncated variant, TPPP/p20, could still bind and bundle MTs^29^. However, a more recent study showed that both N- and C-terminal truncation mutants of TPPP/p25 retain MT binding and bundling activities^30^. The current study aims to further characterize how TPPP/p25 interacts with tubulin and MTs from a mechanistic point of view. This new insight may contribute to a better understanding of the function of TPPP/p25 through its stabilization of physiological microtubule ultrastructures. We address the *in vitro* MT binding and bundling activities of full-length and N- and C-terminally truncated TPPP/p25 by coupling light scattering and electron microscopy (EM) with tubulin copolymerization assays and by quantifying the affinity of the different TPPP/p25 fragments for taxol stabilized MTs. Finally, using Bimolecular fluorescence complementation assays in cells^31^, we demonstrate that the bundling activity of TPPP/p25 is achieved by homotypic protein-protein interactions mediated by the central p25 domain depending on the ability of the protein to bind MTs.

## Materials and Methods

### Plasmids for bacterial expression

The full-length cDNA coding for the human TPPP/p25 (NP_008961.1) was chemically synthesized by GeneArt (Life Technologies), following codon optimization for expression in bacteria. To prepare the various deletion fragments the following primers were used for PCR amplification: ΔN(49) forward 5-gtgatgcgagatctgccatggcactggaagaagcatttcg-3 and reverse 5-cactagaattcctcgagtttgccaccctgaactttctg-3; ΔC(158) forward 5-gtgatgcgagatctgccatggcagataaagcaaaaccg-3 and reverse 5-actagaattcctcgagtgctttggtaacaccgct-3; core forward 5-gtgatgcgagatctgccatggcactggaagaagcatttcg-3 and reverse 5-actagaattcctcgagtgctttggtaacaccgct-3. The PCR products, following purification were ligated first in the pJET1.2/blunt vector (Fermentas) and then the coding regions were inserted in the NcoI-XhoI sites of the pET-23d expression vector coding for 6×HIS terminal tag. The NcoI-XhoI digested inserts were similarly inserted in the pETM11 vector that has been modified to code for chimeras having 6×HIS-GFP tag at the N-terminus of the bacterially expressed proteins (a generous gift of Dr Florent Bernaudat; ESRF Grenoble). The stop codon in the pETM11 TPPP/25 expressing vectors was introduced by point mutagenesis using the following primers for the full length: forward 5′-ggtggtggtggtgctactatttgccaccctgaactttctgatcatagg-3′ and reverse 5′-cctatgatcagaaagttcagggtggcaaatagtagcaccaccaccacc-3′; for the core: forward 5′-tggtggtggtggtgctcctatttgccaccctgaactttc-3′ and reverse 5′-gaaagttcagggtggcaaataggagcaccaccaccacca-3′. All plasmid vectors were sequence verified before use.

### BiFC plasmids

The BiFC plasmids, pBiFC-VN173, pBiFC-VC155 and pBiFC-VN155(I152L), all originated from Dr C-D Hu (Purdue Univeresity, USA) and were purchased from Addgene. To insert the various deletion fragments in either the pBiFC-VN173 or pBiFC-VN155(I152L) the following primers for PCR amplification were used: full length TPPP/p25 forward 5-gcggccgcgaattccatggcagataaagcaaaaccg-3 and reverse 5-gtcgactggtaccgagccaccctgaactttctg-3; ΔN(49) forward 5-gcggccgcgaattccgcactggaagagcatttcg -3 and reverse 5-gtcgactggtaccgagccaccctgaactttctg-3; ΔC(158) forward 5-gcggccgcgaattccatggcagataaagcaaaaccg-3 and reverse 5-gtcgactggtaccgatgctttggtaacaccg-3; core forward 5-gcggccgcgaattccgcactggaagaagcatttcg -3 and reverse 5-gtcgactggtaccgatgctttggtaacaccg-3. To insert the various deletion fragments in the pBiFC-VC155 the following primers were used for PCR amplification; full length forward 5-gcccgaattcggatggcagataaagcaaaaccg-3 and reverse 5-cgccggacgggtaccgccaccctgaactttctg-3; ΔN(49) forward 5-gcccgaattcgggcactggaagaagcatttcg-3 and reverse 5-cgccggacgggtaccgccaccctgaactttctg-3; ΔC(158) forward 5-gcccgaattcggatggcagataaagcaaaaccg-3 and reverse 5-cgccggacgggtacctgctttggtaacaccg-3; core forward 5-gcccgaattcgggcactggaagaagcatttcg-3 and reverse 5-cgccggacgggtacc tgctttggtaacaccg-3. The PCR products, following purification were ligated first in the pJET1.2 vector and then the coding regions were inserted in the EcoRI-KpnI sites of the pBiFC vectors. All resulting plasmids were sequence verified before use.

### Expression and Purification of TPPP/p25 constructs

The pET23d and the pETM11 plasmids coding for the various TPPP/p25 fragments were transformed into competent BL21-(DE3) *Escherichia coli* host cells for protein expression. Cells were grown overnight at 37 °C supplemented with kanamycin (50 μg/mL) until an OD_600_ between 0.5 and 1.0 was obtained. Cells were induced with 0.5 mM IPTG and grown at room temperature for 3 h at 37 °C. Bacteria were harvested by centrifugation, frozen in liquid nitrogen, and stored at −80 °C. Cell pellets were lysed in FastPrep-24 (MP Biomedicals) using sand of Fontainebleau (Prolabo) in a lysis buffer (20 mM Tris (pH 8), 500 mM NaCl, 1 mM DTT, and 5 mM imidazole) supplemented with 25 μl of lysozyme (50 μg/ml), DNase I (20 μg/mL) and Complete EDTA-Free Protease Inhibitor Cocktail Tablets (Roche). Following centrifugation 60 min at 50 000 rpm (Beckmann rotor Ti70, 4 °C) the supernatant was loaded onto a 5 mL Ni-NTA column previously equilibrated with the lysis buffer. Loaded column was further washed with increasing concentrations of imidazole in the lysis buffer and protein fragments were eluted in 500 mM imidazole. Eluted fractions were examined by SDS-PAGE and the most concentrated fractions were pooled together and further concentrated with Centricon filters and then dialyzed against 20 mM Tris (pH 8), 65 mM NaCl, 1 mM DTT. Protein aliquots were frozen in liquid nitrogen, and stored at −80 °C until used. Purified proteins were analyzed by N-terminal sequencing, mass spectrometry, analytical ultracentrifugation (AUC), and HPLC/multi-angle light scattering (MALLS) using the respective platforms housed in the Institut de Biologie Structurale (http://www.isbg.fr). Tag-free protein fragments were prepared from the GFP chimeras following overnight cleavage at 4 °C of the N-terminal GFP tag with HIS-tagged TEV protease (using 1:10 mass ratio of protease to protein-chimeras) leaving only a two amino acid (GA) overhang before the N-terminal sequence of TPPP/p25. Tag-cleaved protein was collected from the flow through of a Ni-NTA column loaded with the digestion reaction and further concentrated as described above.

### Microtubule polymerization assays

MAP-free tubulin from bovine brain was prepared as previously described^32^, in PEM buffer (100 mM PIPES, 1 mM MgCl_2_, 1 mM EGTA, 1 mM GTP) and kept frozen at −80 °C. Taxol stabilized microtubules were assembled from tubulin (50 μmol/L) with paclitaxol (50 μmol/L; SIGMA) for 2 h at 37 °C followed by incubation at room temperature for 3 days and then centrifuged (TLA100.3, 50 krpm for 30 min 30 °C). MT pellets were resuspended in PEM and aliquots were frozen at −80 °C. Following thawing, the MT solutions were further incubated at 37 °C for one hour supplemented with paclitaxol (10 μmol/L). Microtubule polymerization assays were performed in 96-well half-area μclear plates using a 96-well photometer (TECAN) measured at a wavelength of 350 nm. The final test volume was 50 μL prepared from solutions kept at 4 °C. The polymerization into microtubules was followed at 350 nm at 37 °C. Data were plotted using the GRAPHPAD PRISM 6 software. Following incubation with the MAP for 15 min at 37 °C, paclitaxol stabilized MTs were centrifuged (TLA100.3, 50 krpm for 30 min 30 °C). The fluorescence of the supernatants and resuspended pellets were then measured with a CLARIOstar plate reader (BMG LABTECH, GmbH). Data were plotted using the GRAPHPAD PRISM 6 software.

### Electron microscopy

Copolymerization and stoichiometry assays, resistance of complexes at cold temperature and affinity of TPPP/p25 constructs with stabilized MTs were examined by negative-stain electron microscopy on a Tecnai 12 instrument (FEI Eindhoven, The Netherlands) with an LaB_6_ electron source operating at 120 kV. Briefly, 3 μl of samples were loaded on the clean side of carbon on mica (carbon/mica interface), negatively stained with 2% (w/v) uranyl acetate, and air-dried. Images were collected with a SC1000 ORIUS TEM CCD camera (Gatan) with a pixel size of 1.5 Å.

### Cell culture and transfections

HeLa cells were grown in D-MEM (Gibco BRL; Paisley, UK), supplemented with 10% fetal calf serum (Hyclone) in 30 mm petri dishes up to 50% confluence before being transfected with the various BiFC plasmids (0.3 μg of each plasmid) using jetPRIME transfection reagent according to the manufacture’s protocol. 24 h following transfections cells were treated with either nocodazole (Sigma) or vinblastine (generous gift from Dr L. Wilson, (UCSB, USA)) for 3 h before fixing.

### Indirect immunofluorescence microscopy

Cells were fixed 20 min in 2% paraformaldehyde and then permeabilized for 3 min with 0.2% Triton X-100 in PBS before being incubated with primary and stained with the secondary antibodies. The following monoclonal antibodies were used: anti-myc antibody (Covance, Berkeley, CA), anti-HA from Covance (AFC-101P), anti-FLAG from Euromedex (EL1B11), and anti-β-tubulin monoclonal antibodies (clone TUB 2.1; Sigma). The Alexa fluor-488 and -568 conjugated goat anti-mouse secondary antibodies (Invitrogen) were used at 400-fold dilution. DNA was detected with 4’,6-Diamidino-2-phenylindole dihydrochloride (DAPI) with the VECTASHIELD (Vector Laboratories Inc, Burlingame, CA) mounting medium. Images were collected with an inverted Olympus IX81 epifluorescence motorized microscope equipped with a motorized piezo stage (Ludl Electronic Products, USA) and with either a Retiga-SRV CCD camera (QImaging) or a CMOS ORCA Flash4 V2, 4 Megapixels, 16 bits (Hamamatsu Phototnics K.K.) driven by VOLOCITY software (Improvision Ltd, UK) with a binning of 1, using a PlanApo 60×NA1.42 objective (Olympus). Images were processed in Adobe Photoshop CS5 (Adobe). The fluorescence intensity signal in the perinuclear region of BiFC positive cells was measured using the VOLOCITY image analysis software; at least 30 cells for each combination of plasmids were measured, in triplicates. All statistical analysis was performed using the online BiostTGV package (marne.u707.jussieu.fr/biostatgv/).

## Results

**Figure 1:**
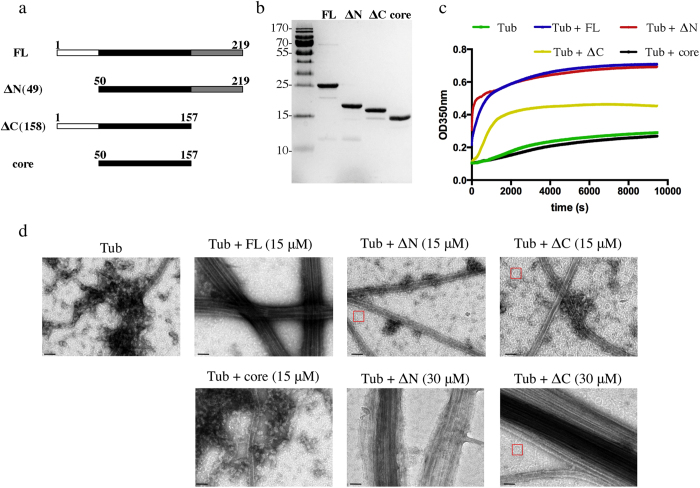
TPPP/p25 induces microtubule polymerization. (**a**) Schematic representation of the various TPPP/p25 fragments used in this study. The ΔN(49) fragments has the first 49 residues deleted whereas the ΔC(158) fragment has residues 158–219 deleted; core fragment lacks both residues 1–49 and 158–219. (**b**) Coomasie-stained gel of bacterially expressed and purified His-tagged TPPP/p25 fragments [FL = full length TPPP/p25; ΔN = ΔN(49); ΔC = ΔC(158)]. (**c**) Light scattering assays (OD_350_ nm) of tubulin solutions (15 μM) in the presence of equimolar concentrations of each of the four TPPP/p25 constructs. (**d**) EM images of microtubules assembled from tubulin solutions (15 μM) in the absence or presence of 15 μM or 30 μM of each of the four different TPPP/p25 fragments (scale bars = 50 nm).

One characteristic property of the TPPP/p25 protein following circular dichroism was shown to be its low alpha helical content^12^,^22^. Consistent with this observation, bioinformatic analysis of the TPPP/p25 amino acid sequence using algorithms to detect folded and disordered protein domains predicted that the protein has disordered N-terminal (first 50 residues) and C-terminal (last 61 residues) regions (Fig. S1)^29^,^33^. Furthermore, the NMR structure of TPPP/p20 (PDB entry 2JRF), another member of the TPPP protein family, revealed unstructured N- and C-termini and a folded central domain composed of five alpha helices^25^. TPPP/p20 has a shorter N-terminus and shares amino acid similarity in the central and C-terminal domains compared to TPPP/p25^34^. We also observed that bacterially expressed and purified full-length TPPP/p25 is proteolytically cleaved into different fragments after prolonged incubation with purified tubulin. N-terminal sequencing and MALDI protein mass spectrometry of the resulting peptides identified protein species truncated at either the N-terminus, C-terminus or both (Fig. S2). In view of these results and of the structural and bioinformatic data, we decided to make three TPPP/p25 deletion constructs, which lack either residues 1–49, C-terminal residues 158–219, or both these regions, denoted ΔN(49), ΔC(158), and “core”, respectively (Fig. 1a). The full-length (FL) and three truncated constructs of TPPP/p25 bear a C-terminal His tag for affinity purification following bacterial overexpression (Fig. 1b). TPPP/p25 has been previously reported to form dimers in solution^35^. However, analysis of all four purified constructs by analytical ultracentrifugation and HPLC-MALLS gave no evidence for the existence of dimers, suggesting that the constructs in our solution conditions are monomeric (data not shown).

In order to study the microtubule TPPP/p25 interaction, we added stoichiometric amounts of the different TPPP/p25 constructs to tubulin, which was at a concentration close to that critical for microtubule self-assembly. In the presence of 1 mM GTP at 37 °C, the addition of either FL protein or ΔN(49) resulted in increased turbidity in light scattering assays (Fig. 1c). In contrast, smaller turbidity changes were observed with the ΔC(158) fragment and no effect was observed upon addition of the core domain. These changes in turbidity suggest that FL and ΔN(49) strongly induce microtubule polymerization, whereas the ΔC(158) has weaker activity and the central folded core none at all.

In order to confirm MT polymerization activity, we monitored in parallel the solution for the presence of MTs by EM. In the control solution containing MAP-free tubulin only a few single MTs were observed. In contrast, consistent with previous reports^12^,^30^, we observed that full length TPPP/p25 stimulated MT assembly and induced MT bundling (Fig. 1d). However, at equimolar ratios of tubulin and either ΔN(49) or ΔC(158) the increased turbidity was associated with limited MT assembly and no MT-bundling activity (Fig. 1d). Instead we observed protein aggregates and abundant rings, which were absent in the control tubulin solution and are characteristic intermediates of MT polymerization as well as depolymerization^36^,^37^,^38^,^39^ (Fig. 1d). However, at a TPPP/p25 : tubulin stoichiometry of 2:1 the increase in turbidity was associated with the presence of MT-bundles induced by either the ΔN(49) or ΔC(158) fragments. We also noted the absence of tubulin rings in the case of ΔN(49), whereas they were present (in addition to the MT bundles) in the case of ΔC(158). Therefore, both N-terminally and C-terminally truncated TPPP/p25 retain the MT bundling activity of the full-length protein, albeit at higher concentrations.

**Figure 2:**
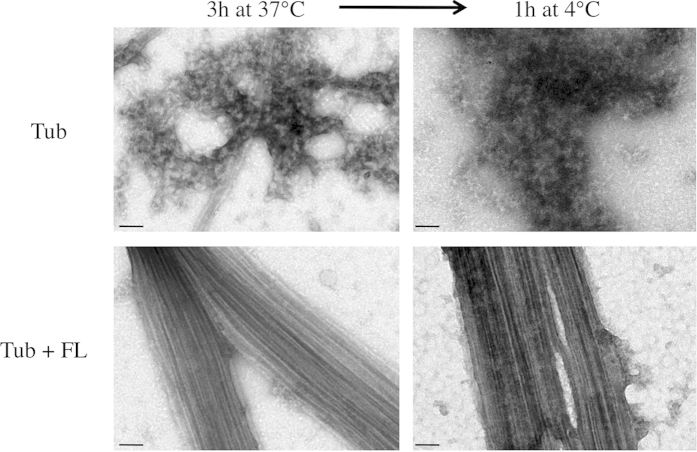
Microtubule bundle cold stability induced by TPPP/p25. Microtubules were assembled from tubulin solutions (15 μM) in the absence or presence of TPPP/p25 fragments (15 μM) to steady state and then the temperature was dropped to 4 °C. EM images of the solutions were taken at the indicated times (scale bars = 50 nm).

We then tested the stability of the TPPP/p25-induced MT bundles to cold treatment. At a TPPP/p25 : tubulin stoichiometry of 1:1, the MT bundles in the presence of full length protein were stable after incubation for 1 h at 4 °C (Fig. 2). In contrast, the single MTs observed in the control were depolymerized and protein aggregates were observed. Therefore, we conclude that TPPP/p25 not only has MT bundling activity but also confers cold stability to MT bundles.

The properties of the C-terminally His-tagged protein appeared to be similar to the protein isolated from the native source^12^. However, in order to address the issue of the impact of the tag on the TPPP/p25 MT-binding characteristics we also carried out experiments with the full length and the core of TPPP/p25 following proteolytic removal of the His tag. The tag-free FL TPPP/p25 stimulated both MT assembly and bundling activities as detected by turbidity measurements and EM respectively, whereas the core showed no such activity even at higher concentrations (Fig. S3, panels A and B). Compared to the His-tagged protein the tag-free protein was less efficient in inducing the polymerization of tubulin (stoichiometric ratios 1:1 and 1:2, respectively).

**Figure 3:**
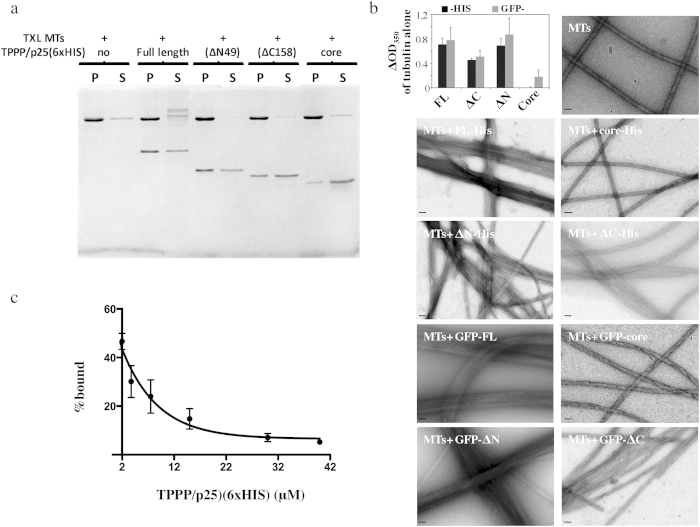
Microtubule binding properties of TPPP/p25. (**a**) Taxol stabilized microtubules (2 μM) were incubated in the presence of 2 μM of each of the different TPPP/p25 fragments (FL, ΔN, ΔC and core) for 15 min before centrifugation. Coomassie-stained gel of microtubule bound and unbound TPPP/ p25 fragments present in the pellets and in the supernatants, respectively. (**b**) Bar graph showing the changes in turbidity of tubulin solutions (15 μM) in the presence of stoichiometric amounts of the different TPPP/ p25 fragments. The OD_350_ of control tubulin solutions at mass steady state were subtracted from the tubulin solutions assembled in the presence of the different TPPP/ p25 fragments tagged with either 6×HIS at their C-termini or GFP at the N-termini. Data were compared using the Mann Whitney test (p values for the HIS- vs the GFP- tagged fragments were 1, 0,666, 0,667 and 0,333 for FL, ΔN, ΔC, and core respectively; p values for the FL, or ΔN, or ΔC fragments compared to the core were 0,028, 0,2 and 0,57, respectively; n = 4). EM images of taxol stabilized microtubules (2 μM) incubated with bacterially expressed and then purified C-terminal His- or GFP-tagged TPPP/p25 fragments at equimolar ratios (scale bars = 50 nm). (**c**) His tagged TPPP/p25 competes with GFP-tagged TPPP/p25 for MT binding. Taxol stabilized MTs were first co-incubated with 2 μM of 6×HIS-FL and 2 μM GFP-FL-TPPP/p25 and then with increasing concentrations of 6×HIS-FL. Each data point represents the mean ± SD from three independent experiments.

In order to analyze the MT binding affinity of TPPP/p25 and its various fragments, we exploited a MT pelleting assay in which the amount of TPPP/p25 bound to taxol MT pellets is measured. We carried out preliminary experiments with all the His-tagged fragments of TPPP/p25 at stoichiometric 1:1 ratios with taxol stabilized MTs (Fig. 3a). The MT-pelleting assays showed that indeed the full length as well as the ΔN(49) and ΔC(158) bind to MTs whereas the core had very limited MT binding activity. The binding of the tag-free FL TPPP/p25 to taxol stabilized MTs were qualitatively similar to His-tagged FL TPPP/p25 whereas the tag-free core did not exhibit any binding to taxol MTs (Fig. S3, panels C and D).

Further attempts to quantify the MT-bound TPPP/p25 by scanning the gels or blots gave erratic results in our hands. We therefore switched to a fluorescence measurement assay using TPPP/p25 constructs that were N-terminally tagged with GFP. First, we assessed whether the two sets of constructs (GFP- and His-tagged) were equivalent in their ability to bind and bundle MTs. We carried out light scattering assays of tubulin solutions in the presence of GFP-tagged TPPP/p25 fragments, similar to those carried out with the His-tagged (Fig. 1c) and tag-free proteins (Fig. S3, panels C and D). At mass steady state, we calculated the changes in OD_350_ due to addition of the various protein fragments by subtracting the OD_350_ observed of the tubulin solution alone. The changes of turbidity in tubulin solutions obtained with the GFP-tagged were similar to those obtained with the His-tagged TPPP/p25 fragments. Most importantly, EM imaging of taxol stabilized MTs incubated with either His- or GFP- tagged TPPP/p25 were indistinguishable. The bundling activities of GFP-tagged full length, ΔN(49) and ΔC(158) proteins were retained whereas GFP-core had no MT bundling activity (Fig. 3b). Finally, in order to determine if the two differentially tagged proteins are equivalent we carried out competition assays using the fluorescent binding assay. Indeed, the His-tagged FL protein could displace the equivalent GFP-tagged protein for MT binding (Fig. 3c). We therefore concluded that the N-terminally GFP-tagged and C-terminally His-tagged TPPP/p25 fragments were equally functional in *in vitro* MT binding assays. Similarly to the His-tagged TPPP/p25, the tag-free protein competed for MT binding of the GFP-tagged TPPP/p25 (Fig. S4). Based on this result any major influence of the tag (s) on the MT binding properties of TPPP/p25 can be excluded.

**Figure 4:**
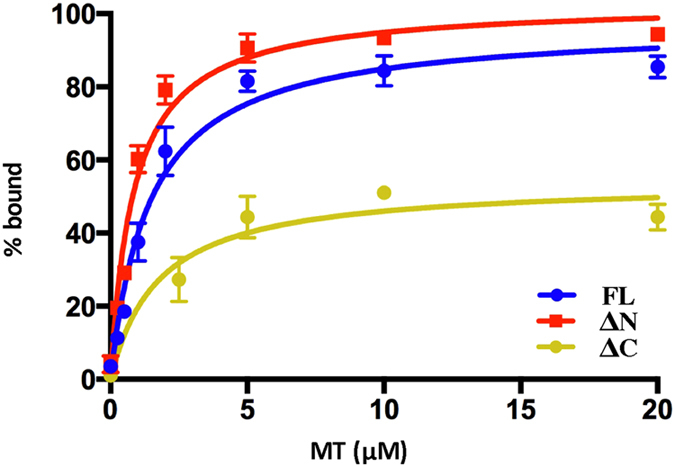
Quantitative analysis of the TPPP/p25 binding interaction to taxol stabilized microtubules. Binding curves with 1.5 μM GFP-tagged TPPP/p25 (WT or ΔN, ΔC) at increasing MT concentrations. Each data point represents the mean ± SD from three independent experiments.

We then proceeded to quantify the fluorescence signal of the bound GFP-TPPP/p25 fragments from the MT pellets and the unbound in the supernatant fraction after incubation of 1.5 μM TPPP/p25 with increasing concentrations of taxol stabilized MTs (Fig. 4). Following this experimental approach the apparent K_d_ for the three TPPP/p25 fragments was determined to be 1.44 ± 0.26 μM for the full length protein, 0.86 ± 0.15 μM for ΔN(49) and 1.75 ± 1.03 μM for the ΔC(158) (Table 1). Given the observation that MTs bundle in the presence of TPPP/p25 the apparent K_d_ is likely to be a composite of MT binding and TPPP/p25 dimerization or oligomerization. A notable difference was observed in the Bmax for the C-terminal deleted fragment ΔC(158), which was 50% of that of the full length or ΔN(49) fragment (Table 1).

**Figure 5:**
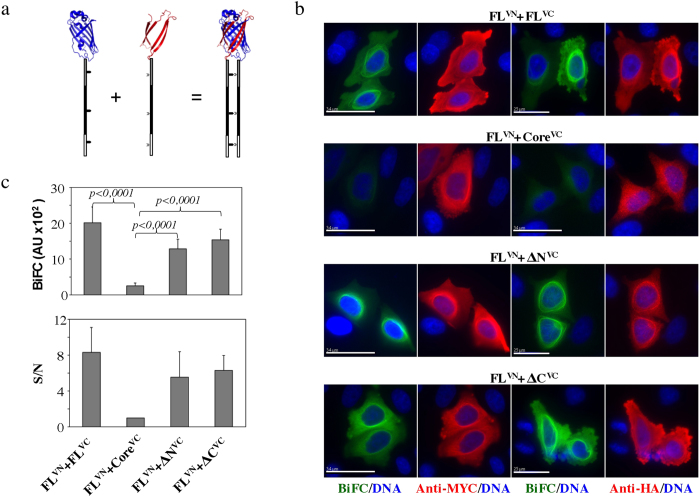
TPPP/p25-TPPP/p25 interactions in cells detected by BiFC. (**a**) Schematic representation of the generation of fluorescence based on the complementation between the N- and C-terminal domains of Venus fluorescent protein fused to proteins capable of interacting with each other. (**b**) BiFC signal generation in cells that were co-transfected with the myc-tagged FL fused to VN155(I152L), indicated as FL^VN^ and either HA-tagged FL- or ΔN-, ΔC- and core- fused to the VC155, indicated as FL^VC^ or ΔN^VC^, or ΔC^VC^ or core^VC^, respectively. Co-expression of the two chimeras was confirmed by immunofluorescence microscopy using antibodies recognizing either the myc- or the HA- tags. (**c**) Fluorescence intensities of Venus-based BiFC assays in cells transfected as described in panel (**b**). Each bar represents the mean ± SD from three independent experiments (at least 30 BiFC-positive cells measured for each count). Data were compared using the Mann Whitney test (n = 100, 113, 110 and 95 for FL^VN^-FL^VC^, FL^VN^-core^VC^, ΔN^VN^-core^VC^ and ΔC^VN^-core^VC^, respectively). The signal to noise (S/N) ratio was estimated by dividing the fluorescence intensity from the positive interaction (FL^VN^-FL^VC^; FL^VN^-ΔN^VC^; FL^VN^-ΔC^VC^) by that from the negative interaction (FL^VN^-core^VC^) (scale bars = 17 μm).

A number of MAPs, have the ability to induce MT bundles under certain conditions. The bundling activity of PRC1 and its homologues Ase1 and MAP65-1 were shown to be due to dimer formation following MT-binding^7^. Similarly, tau has also been reported to oligomerize along MTs^40^. In order to investigate the MT-dependent self-association of TPPP/p25 and map the domains responsible for this interaction in cells we employed a Bimolecular Fluorescent Complementation (BiFC) assay^41^. For BiFC, the N-terminal and the C-terminal domains of a fluorescent protein (e.g., GFP, Venus) are each fused separately to two partners hypothesized to interact with each other^42^. If the two partners indeed interact, then upon interaction the two domains of the split fluorescent protein will come into close proximity and fold into the native structure, which will fluoresce upon excitation. We have chosen to use the Venus fluorescent protein since it has superior BiFC properties than GFP (Fig. 5a)^43^. A necessary step for the BiFC assay is to generate a negative control in order to distinguish the true BiFC signal from the non-specific BiFC signal generated by the intrinsic tendency of the two fluorescent protein domains to complement each other^44^. Our *in vitro* MT binding data indicated that the core domain is not interacting with MTs and therefore we hypothesized that in the absence of MT binding it would have a low tendency for self association and therefore should produce low BiFC signal. Furthermore, we used two different Venus N-terminal domains, one comprising the first 173 residues and the other comprising the first 155 residues carrying a I152L mutation. Both Venus N-terminal fragments are able to complement the Venus C-terminal domain starting from residue 155. The VN155(I52L) fragment has been reported to reduce the nonspecific BiFC and therefore to produce higher BiFC signal to noise ratios^45^.

We fused all the TPPP/25 fragments with the various pBiFC vectors and carried transient co-transfections in cells of the pBiFC-myc-FL-VN155(I152L) in combination with either pBiFC-HA-FL-VC155, pBiFC-HA-ΔN-VC155, pBiFC-HA-ΔC-VC155 or pBiFC-HA-core-VC155 constructs. A strong perinuclear filamentous BiFC signal was detected for the pairs FL^VN^-FL^VC^, FL^VN^-ΔN^VC^ and FL^VN^-ΔC^VC^ whereas the pair FL^VN^-core^VC^ gave a low BiFC signal (Fig. 5b). We measured the BiFC signal generated from the different plasmid combinations and by defining the low signal measured from the FL^VN^-core^VC^ pair as noise, we observed that there was an 8-fold increase in the BiFC signal obtained by the FL^VN^-FL^VC^ pair (Fig. 5c). An increased signal-to-noise (S/N) ratio was observed in the cases of the FL^VN^-ΔN^VC^ and FL^VN^-ΔC^VC^ pairs. The expression of both constructs in cells was also verified by carrying indirect immunofluorescence microscopy using antibodies detecting the two different tags, myc and HA, respectively. The tag signal colocalized with the BiFC signal in the perinuclear filaments and in general was more diffused in the cytoplasm than the BiFC signal (Fig. 5b). In the case of the FL^VN^-core pair the FL protein (detected by anti-myc) had a filamentous staining as expected, whereas the low BiFC signal was diffused, as was the staining of the expressed core (detected with anti-HA).

**Figure 6:**
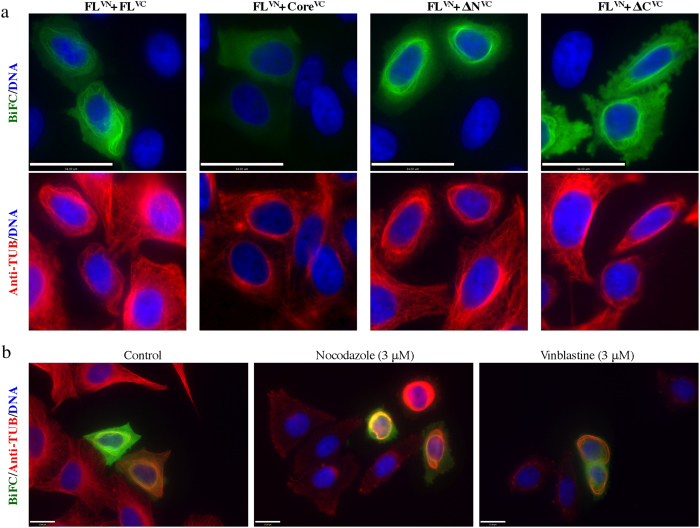
TPPP/p25-TPPP/p25 interactions in cells detected by BiFC on microtubule bundles. (**a**) The generated BiFC signal resulting from the FL^VN^-FL^VC^, FL^VN^-ΔN^VC^, FL^VN^-ΔC^VC^ complex formation colocalizes with perinuclear microtubule bundles. Note the absence of BiFC signal despite the presence of microtubule bundles due to the expression of the FL^VN^ in cells (scale bars = 34 μm). (**b**) Microtubule bundles in cells transfected with the wild type TPPP/p25 are resistant to nocodazole and vinblastine treatment (scale bars = 17 μm).

The perinuclear filamentous BiFC colocalized with dense perinuclear MT bundles (Fig. 6a). Expression of the FL^VN^-FL^VC^, FL^VN^-ΔN^VC^ and FL^VN^-ΔC^VC^ pairs induced the formation of the MT-bundles. Although the FL-core pair did not produce BiFC, there were still perinuclear MT bundles formed because of the bundling activity of the full-length protein overexpressed in cells. It is worth mentioning that single plasmid transfections gave the same patterns of localization of the various constructs, eliminating the possibility that the dimerization of Venus protein was the cause of the MT bundling (Fig. S5). Similar MT bundles were observed previously when GFP-tagged TPPP/p25 was expressed in cells^26^,^28^. Interestingly, the perinuclear MT bundles formed due to the expression of the FL^VN^-FL^VC^ pair were resistant to MT depolymerizing drugs such as nocodazole and vinblastine. In non-transfected cells the entire MT network was depolymerized in the presence of nocodazole, whereas in the BiFC positive cells the MT bundles resisted depolymerization. In vinblastine treated cells tubulin paracrystals were observed in nontransfected cells^46^, whereas in the BiFC positive cells there were no tubulin paracrystals formed, probably due to the lack of enough soluble tubulin since the perinuclear MT bundles resisted depolymerization.

**Figure 7:**
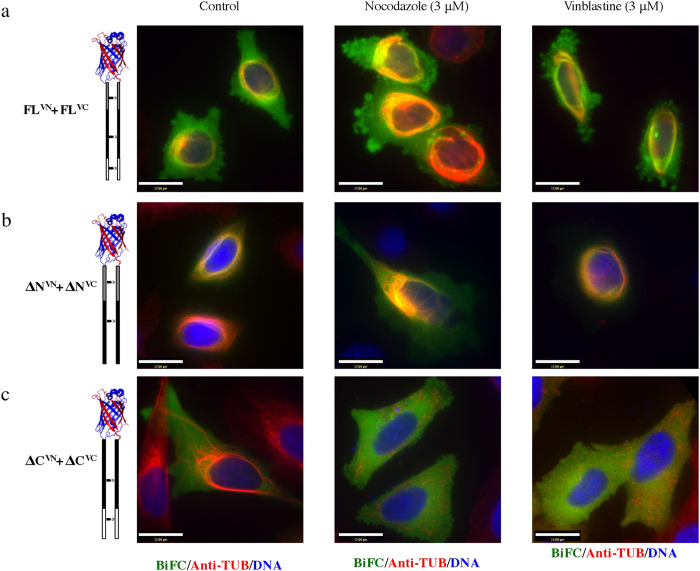
Differential stability of the TPPP/p25 induced microtubule bundles in cells. Cells were co-transfected with either FL^VN^-FL^VC^ (**a**), ΔN^VN^-ΔN^VC^ (**b**), ΔC^VN^-ΔC^VC^ (**c**) and then incubated with nocodazole or vinblastine. Only the ΔC^VN^-ΔC^VC^ microtubule bundles were sensitive to both microtubule inhibitors (scale bars = 17 μm).

The MT bundle stability in the presence of MT depolymerizing drugs was further tested in cells expressing pairs of the different TPPP/p25 fragments. The MT bundles generated by expression of the ΔN-ΔN pair exhibited similar stability as the FL^VN^-FL^VC^ pair; the generated BiFC signal and the perinuclear MT bundles were insensitive to nocodazole and vinblastine induced depolymerization (Fig. 7). However, the MT bundles generated due to the expression of the ΔC^VN^-ΔC^VC^ pair were sensitive to depolymerization by both drugs. The data suggest that the ΔC(158) may have lower MT stabilizing properties than the FL and ΔN(49) proteins.

## Discussion

Our presented study aimed at investigating the microtubule binding and bundling activities of the human TPPP/p25 protein found in neuronal and glial inclusion protein aggregates in patients suffering of Parkinson’s disease. The precise mechanism by which TPPP/p25 contributes to toxicity in Parkinson’s disease is unknown, however, it is thought that a loss of native function and/or a gain of toxic function are implicated. To date, the only biological function attributed to native TPPP/p25 is related to its ability to bind and bundle MTs, offering an explanation as to how TPPP/p25 may influence the plasticity of MT networks in neuronal cells. Mapping of the MT binding site through deletion analysis revealed that the protein has at least two MT binding sites, one at the N-terminus (residues 1–49) and the other at the C-terminus (residues 158–219); the central core domain lacked any MT binding properties. Both N- and C-terminal regions are intrinsically disordered and highly basic (pI 10.12 and 10.15 respectively) and therefore one can suggest that the binding of both domains to microtubules is achieved through electrostatic interactions with the acidic C-terminal tails of tubulin extending from the MTs (Fig. S1B). Our data with tubulin concentrations close to the critical concentration of assembly show that although the full-length protein can assemble and bundle microtubules at a MAP: tubulin molar ratio of 1:1, both the N- and C- deleted fragments of TPPP/p25 have lost their bundling activity. According to increased light scattering observed, both the N- and the C-terminal truncations of TPPP/25 lower the critical tubulin concentration required for microtubule assembly. However, the turbidity increase was also associated with the presence of abundant tubulin rings and few MTs of various sizes seen by EM. Interestingly such rings are detected after cold depolymerization as well as in the presence of MT depolymerizing, drugs, peptides and kinesin motor proteins^47^,^48^,^49^,^50^. The deleted fragments may have the ability to induce or trap the tubulin polymerization/depolymerization intermediates at low tubulin concentrations. Further sophisticated imaging analysis is needed to determine whether TPPP/p25 is situated in the internal or external surface of the rings. Higher stoichiometric amounts of TPPP/p25 N- and C-terminal fragments resulted in MT bundles similar in morphology to those observed with the full-length protein. Therefore, for optimal function of the TPPP/p25 both domains are needed. The shorter N-terminus of the TPPP/p20 compared to TPPP/p25 suggests that the two proteins may have different functions in cells, serving different roles during the cell cycle.

During the course of our experiments we confirmed that the tag-free protein has a similar impact on MT assembly and bundling, even though at higher ratios of TPPP/p25 to tubulin compared to the His- and GFP-tagged proteins. Similar impact of the tag on the microtubule associated protein EB1 has been reported previously^51^. Most importantly though, the tag-free as well as the tagged core domain have no impact on MT assembly and both lack bundling activity. Because of the intrinsically unstable nature of the TPPP/p25, the presence of the His and GFP tag in the chimeric protein may enrich a population from all the different ensembles of the protein structure that are more favorable for MT binding. Therefore, the TEV protease mediated cleavage of the tag may lead to further destabilization of the protein. However, previous reports have documented that the MT assembly stimulation and bundling activities of the protein isolated from the native source are retained 12 as well as the ^1^H-NMR spectra of the native protein as well as the recombinant human TPPP/p25a were similar^19^, therefore the impact of the tag may have only quantitative and not qualitative impact on the protein’s properties. One can suggest that putative electrostatic interactions responsible for the binding of TPPP/p25 to MTs may be enhanced through the presence of the tag.

One prominent property of the TPPP/p25 is its ability to bundle MTs. Other MT associated proteins such as PRC1 and its family members^7^, as well as Ndc80^52^, MAP2 and tau are also shown to induce MT bundling^53^. In the case of PRC1/MAP65-1 induced MT bundles, extensive intermolecular bridges connecting two antiparallel MTs are seen by EM. The PRC1/MAP65-1 bridges are formed by PRC1/MAP65-1 dimers^6^,^54^, with each monomer bound to a different MT. Similar interbridges are seen for Ndc80^52^ and MAP2^53^. However, such TPPP/25 interbridges connecting the crosslinked MTs were not seen by EM, but this may be due to the small size of the protein, which is compounded by the fact that the N- and C-terminal regions are intrinsically disordered, leaving only a small part of the protein folded. In the case of tau-induced MT bundles, a tau dimer is thought to be responsible for MT bundle formation. Tau dimers are formed by an electrostatic “zipper” formed by salt bridges between opposite charged domains of the N-termini of the two tau molecules, each one bound to opposing MTs through their C-termini^40^. Alternating charge distributions exist also in TPPP/p25 (Fig. S1B) and so an electrostatic “zipper” interaction mechanism would also be feasible for TPPP/p25 –mediated MT bundling.

**Figure 8:**
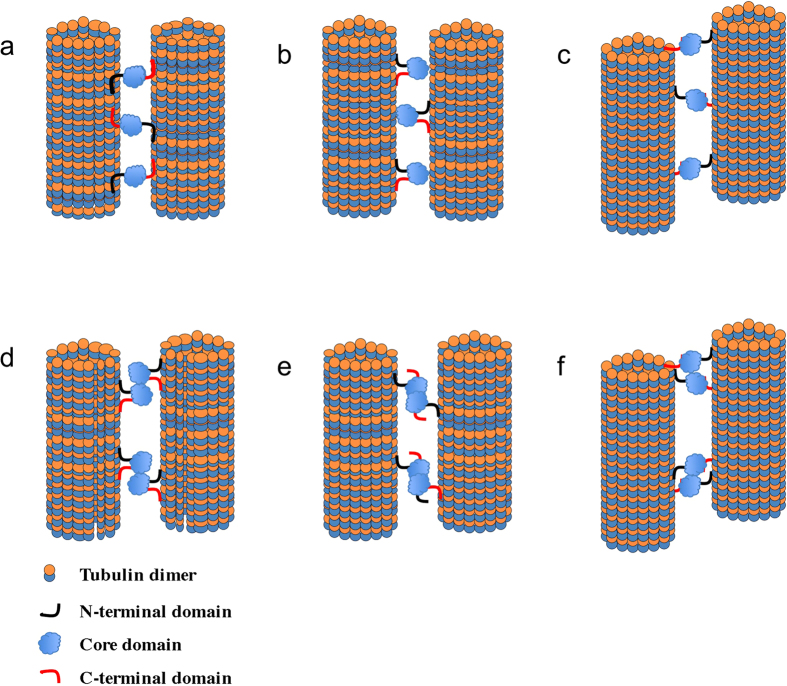
Schematic representation of the possible interaction modes of TPPP/p25 resulting in microtubule binding and bundling. The various elements and distances are not drawn to scale.

The presence of two MT binding sites on TPPP/p25 provides an obvious model for how one molecule of TPPP/p25 can crosslink two MTs, with each binding site interacting with a different MT (Fig. 8, models A and C). Alternatively, one of the sites on TPPP/p25 might bind a tubulin site which is masked when tubulin is assembled and thus only accessible at the tips of the MTs (Fig. 8, model C). However, our deletion analysis showed that both N-terminal and C-terminal deleted TPPP/p25 fragments retain their ability to bundle MTs. An alternative model is that TPPP/p225 may interact with 3 different sites (Fig. 8, model B). Our current domain analysis though, does not provide evidence for the presence of a third binding site. Therefore, we propose a dimer, like that proposed for the PRC1/MAP65-1 or the tau MT induced bundling^5^,^7^. These models are based on the ability of the MAP to form dimers that could crosslink two MTs. The issue of TPPP/p25 dimer formation has been proposed previously, and depending on GTP concentration and crosslinking conditions, TPPP/p25 dimers have been detected^35^. Our biophysical analysis of all the fragments though did not support the formation of TPPP/p25 dimers in solution. However, one could propose a conditional protein-protein interaction that would depend on MT binding (Fig. 8, models D, E and F). Full-length and the N-and C-terminal deleted fragments in our hands had very similar K_d_ for MT binding. However, the C-terminal deleted fragment exhibited a Bmax that was 50% lower than the full length and N-terminal deleted fragment. One hypothesis that can explain this difference is that the N- and C-termini bind to different sites and that the tubulin binding site for the C-terminal domain is masked in taxol stabilized MTs. One possible binding site for the C-terminus of TPPP/p25 may be at the tips of the MTs (Fig. 8, model F). Further analysis of the possible MT tip binding properties of the TPPP/p25 is needed.

The MT driven protein-protein interaction properties of TPPP/p25 were further validated in our current study by BiFC studies. The BiFC assay by ectopic expression of the different protein fragments in cells gave us clear indication that protein-protein interactions indeed take place along MT bundles. Previous studies have shown that expression of GFP-TPPP/p25 induces intracellular perinuclear MT bundles that were resistant to depolymerization by MT destabilizing drugs. The BiFC signal observed in cells, resulting from interactions between full-length and N- or C-terminally truncated versions of TPPP/p25 are totally consistent with the previous results. Additionally, BiFC shows that indeed the protein-protein interactions between the different TPPP/p25 fragments depend on the presence of the core domain. However, the core domain alone does not bind MTs *in vitro* and in cells is not capable of generating a BiFC signal when expressed together with either full-length TPPP/p25 or truncation mutants. Given that TPPP/p25 appears to be a monomer in solution, self-association is likely triggered by the interaction with MTs. Indeed, one hypothesis is that the central alpha helical domain dimerizes but that the dimerization interface is masked by the N- or C-terminal region and becomes exposed upon MT association. Further biochemical and structural studies are needed to confirm the role of the core central domain of TPPP/p25 in the protein function.

## Additional Information

**How to cite this article**: DeBonis, S. *et al.* Self protein-protein interactions are involved in TPPP/p25 mediated microtubule bundling. *Sci. Rep.* **5**, 13242; doi: 10.1038/srep13242 (2015).

## Supplementary Material

Supllementary Data

## Figures and Tables

**Table 1:** Parameters of full length and deleted forms of TPPP/p25 binding to taxol stabilized microtubules.

	Full length	ΔN(49)	ΔC(158)
Kd	1.44 ± 0.26	0.86 ± 0.15	1.75 ± 1.02
Bmax	97.13 ± 4,9	103 ± 4,6	54.05 ± 4,9
