# Supplementary material for: Self protein-protein interactions are involved in TPPP/p25 mediated microtubule bundling
Source: Sci Rep. 2015 Aug 20;5:13242. doi: 10.1038/srep13242 (PMC4542545; doi:10.1038/srep13242)
Supplement: Supllementary Data [file srep13242-s1.pdf]

Self protein-protein interactions are involved in TPPP/p25 mediated microtubule bundling

Salvatore DeBonis[3,1,2,§], Emmanuelle Neumann[3,1,2§], Dimitrios A. Skoufias[3,1,2*]

[1]Université de Grenoble Alpes, [2]CNRS, [3]CEA, IBS, F-38044 Grenoble, France

Supplementary Figures Legends

Figure S1 (a) Folding predictions of human TPPP/p25 according to the FOLDINDEX software, which predicts whether a given protein sequence is intrinsically unfolded. The N-and C-terminal domains are estimated to have a high probability of being intrinsically unfolded (red) in contrast to the central core domain (green) [33]. **(b)** Charge distribution plot of TPPP/p25 showing that the N-terminus and C-terminus are positively charged. The core domain has alternating charges favoring a proposed "electrostatic zipper" of protein-protein interaction mode.

Figure S2 (a) Coomasie blue staining of full length TPPP/p25 and the supernatant after centrifugation following long term incubation (longer than 24h) with taxol stabilized MTs. **(b)** The proteolytic fragments were analyzed by N-terminal sequencing and the molecular masses were determined by MALDI mass spectrometry. **(c)** The corresponding N-terminal sequences are depicted in red in the amino acid sequence of full length TPPP/p25.

Figure S3 MT binding properties of tag-free TPPP/p25. **(a)** Light scattering assays (OD_{350} nm) of tubulin solutions (15 μM) in the presence of His-tagged full length (FL) and tag cleaved full length ($FL^{HISFree}$) and $core^{HISFree}$ domain of TPPP/p25 (ratios of tubulin to TPPP/p25 are shown in parentheses). **(b)** EM images of microtubules assembled from tubulin solutions (15 μM) in the presence of FL and tag-cleaved FL and tag-cleaved core domain of TPPP/p25 (ratios of tubulin to TPPP/p25 are shown in parentheses). Scale bars in the left column of images correspond to 0,5 μm; in the right column to 0,2μm). **(c)** Taxol stabilized microtubules (2 μM tubulin) were incubated in the presence of 12 μM of either tag-cleaved FL or core domain of TPPP/p25 for 15 min

before centrifugation. Coomassie-stained gel of microtubule bound and unbound TPPP/ p25 fragments present in the pellets and in the supernatants, respectively. **(d)** EM images of taxol stabilized microtubules incubated with bacterially expressed and purified tag-cleaved TPPP/p25 fragments (ratios of tubulin to TPPP/25 are shown in parentheses) Scale bars correspond to 0,2μm.

Figure S4 Tag-cleaved TPPP/p25 competes with GFP-tagged TPPP/p25 for MT binding. Taxol stabilized MTs were first co-incubated with 2 μM of tag-cleaved FL and 2 μM GFP-FL-TPPP/p25 and then with increasing concentrations of tag-cleaved FL. Each data point represents the mean ± SD from three independent experiments.

Figure S5 Cells were transfected with either HA-full length **(a)**, ΔC(158) **(b)**, ΔN(49) **(c)** or core -TPPP/p25-VC173 **(d)**. Cells were then fixed and stained for indirect immunofluorescence microscopy using a monoclonal antibody recognizing the HA-tag. Perinuclear bundles are detected in cells transfected with the full length, ΔN(49) and ΔC(158) whereas a diffuse cytoplasmic staining is observed in cells expressing the core.

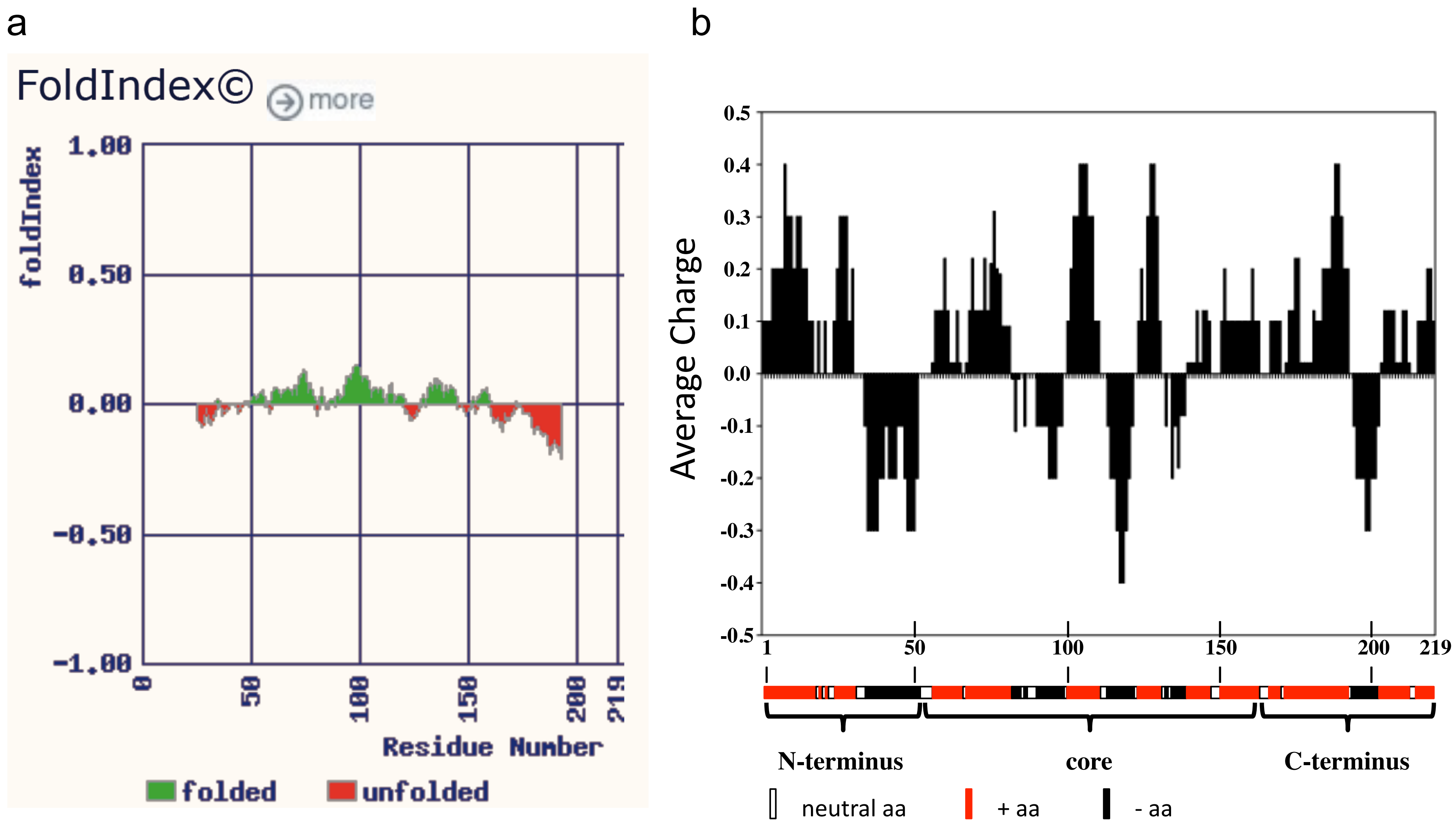
a
FoldIndex©
more
foldIndex
1.00
0.50
0.00
-0.50
-1.00
0
50
100
150
200
219
Residue Number
folded
unfolded
b
Average Charge
0.5
0.4
0.3
0.2
0.1
0.0
-0.1
-0.2
-0.3
-0.4
-0.5
1
50
100
150
200
219
N-terminus
core
C-terminus
neutral aa
+ aa
- aa

a

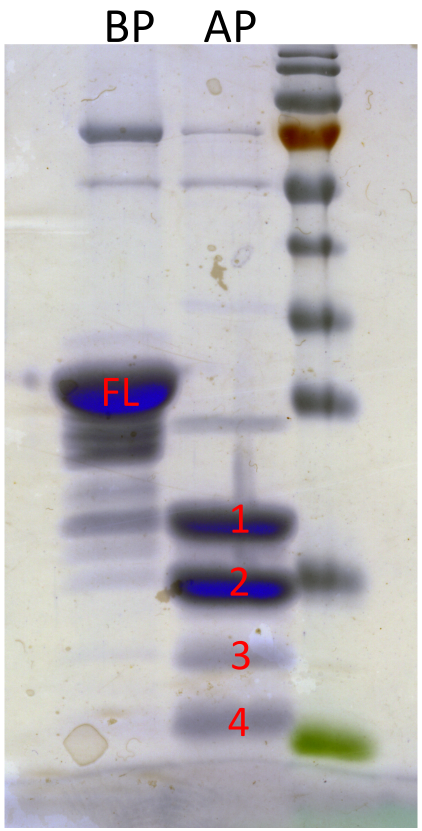

b

SDS-PAGE hTPPP/25 peptide	N-term sequence	Peptide Mass (Da) (MALDI)
FL	ADKAKP	23 694
1	ADKAKP	18580
2	SLESEGAG	15476
3	AVHGDARA	8078
4	SKIKGKSX(72%)<br>ADKAKPAK (22%)<br>EQFQEALE (6%)	5904

c

2 32
MADKAKPAKAANRTPPKSPGDPSKDRAAKRLSLESEGAGEGAAASPELSALEEAFRRF
59 101
AVHGDARATGREMHGKNWSKLCKDCQVIDGRNVTVTDVDIVFSKIKGKSCRTITF
114
EQFQEALEELAKKRFKDKSSEEAVREVHRLIEGKAPIISGVTKAISSPTVSRLTDTTKF

TGSHKERFDPSGKGKGKAGRVDLVDESGYVSGYKHAGTYDQKVQGGK

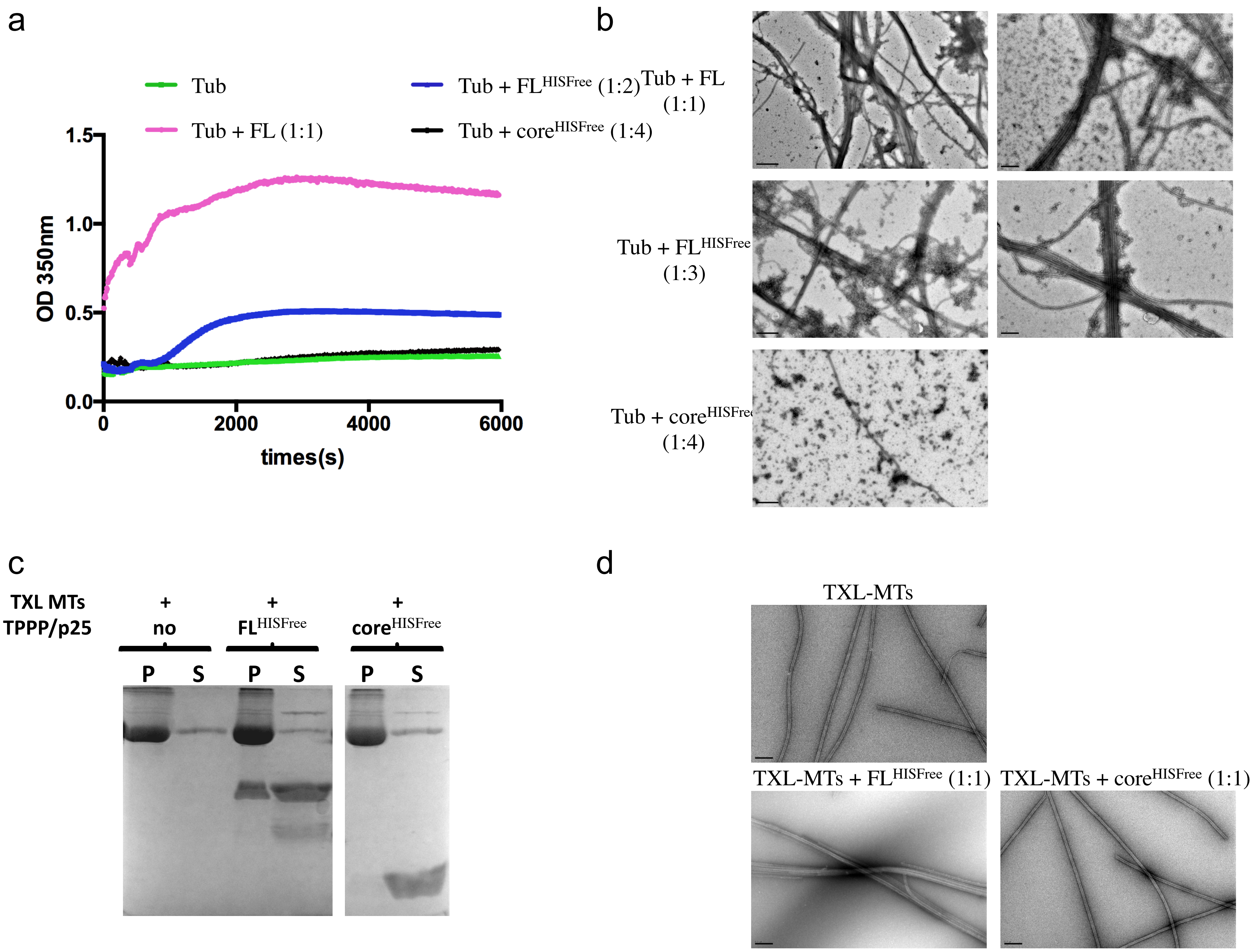

a
Tub
Tub + FL (1:1)
Tub + FL^{HISFree} (1:2)
Tub + core^{HISFree} (1:4)
OD 350nm
1.5
1.0
0.5
0.0
0
2000
4000
6000
times(s)
b
Tub + FL
(1:1)
Tub + FL^{HISFree}
(1:3)
Tub + core^{HISFree}
(1:4)
c
TXL MTs
TPPP/p25
+
no
+
FL^{HISFree}
+
core^{HISFree}
P S P S P S
d
TXL-MTs
TXL-MTs + FL^{HISFree} (1:1)
TXL-MTs + core^{HISFree} (1:1)

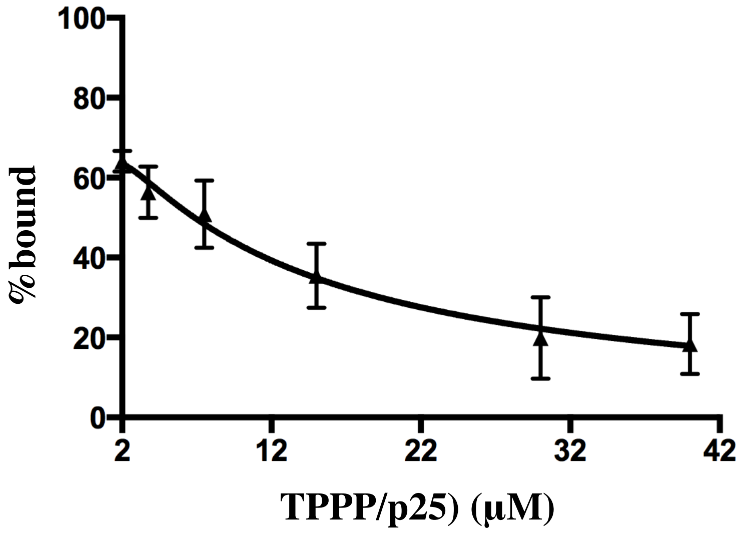
100
80
60
40
20
0
% bound
2
12
22
32
42
TPPP/p25) (μM)

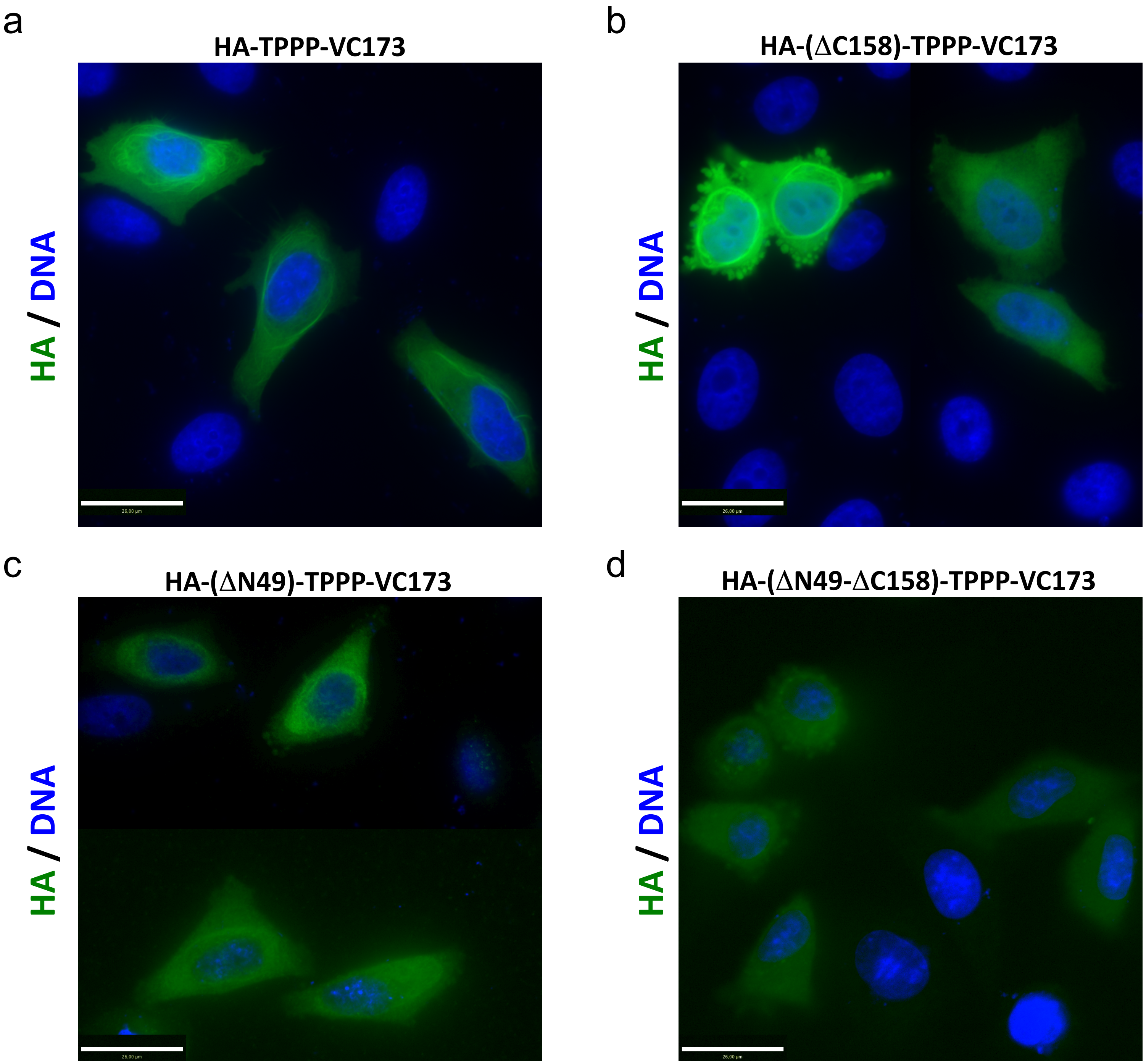
a
HA-TPPP-VC173
HA / DNA
b
HA-(ΔC158)-TPPP-VC173
HA / DNA
c
HA-(ΔN49)-TPPP-VC173
HA / DNA
d
HA-(ΔN49-ΔC158)-TPPP-VC173
HA / DNA
